# Slow-Wave Activity in the S1HL Cortex Is Contributed by Different Layer-Specific Field Potential Sources during Development

**DOI:** 10.1523/JNEUROSCI.1212-19.2019

**Published:** 2019-11-06

**Authors:** Tania Ortuño, Victor J. López-Madrona, Julia Makarova, Silvia Tapia-Gonzalez, Alberto Muñoz, Javier DeFelipe, Oscar Herreras

**Affiliations:** ^1^Departamentos de Neurociencia Traslacional, y; ^2^Neurobiología Funcional y de Sistemas, Instituto Cajal–CSIC, Madrid 28002, Spain,; ^3^Laboratorio Cajal de Circuitos Corticales, Centro de Tecnología Biomédica, Universidad Politécnica de Madrid, Campus Montegancedo, Madrid 28223, Spain,; ^4^Instituto de Neurociencias–CSIC, Universidad Miguel Hernández, San Juan de Alicante 03550, Spain, and; ^5^Departamento de Biología Celular, Universidad Complutense, Madrid 28040, Spain

**Keywords:** cortex, development, field potential, laminar activity, somatosensory hindlimb cortex, spatial discrimination

## Abstract

Spontaneous correlated activity in cortical columns is critical for postnatal circuit refinement. We used spatial discrimination techniques to explore the late maturation of synaptic pathways through the laminar distribution of the field potential (FP) generators underlying spontaneous and evoked activities of the S1HL cortex in juvenile (P14–P16) and adult anesthetized rats. Juveniles exhibit an intermittent FP pattern resembling Up/Down states in adults, but with much reduced power and different laminar distribution. Whereas FPs in active periods are dominated by a layer VI generator in juveniles, in adults a developing multipart generator takes over, displaying current sinks in middle layers (III–V). The blockade of excitatory transmission in upper and middle layers of adults recovered the juvenile-like FP profiles. In addition to the layer VI generator, a gamma-specific generator in supragranular layers was the same in both age groups. While searching for dynamical coupling among generators in juveniles we found significant cross-correlation in ∼one-half of the tested pairs, whereas excessive coherence hindered their efficient separation in adults. Also, potentials evoked by tactile and electrical stimuli showed different short-latency dipoles between the two age groups, and the juveniles lacked the characteristic long latency UP state currents in middle layers. In addition, the mean firing rate of neurons was lower in juveniles. Thus, cortical FPs originate from different intra-columnar segments as they become active postnatally. We suggest that although some cortical segments are active early postnatally, a functional sensory-motor control relies on a delayed maturation and network integration of synaptic connections in middle layers.

**SIGNIFICANCE STATEMENT** Early postnatal activity in the rodent cortex is mostly endogenous, whereas it becomes driven by peripheral input at later stages. The precise schedule for the maturation of synaptic pathways is largely unknown. We explored this in the somatosensory hindlimb cortex at an age when animals begin to use their limbs by uncovering the laminar distribution of the field potential generators underlying the dominant delta waves in juveniles and adults. Our results suggest that field potentials are mostly generated by a pathway in deep layers, whereas other pathways mature later in middle layers and take over in adults. We suggest that a functional sensory-motor control relies on a delayed maturation and network integration of synaptic connections in middle layers.

## Introduction

Evolving patterns of electrical activity correlate with structural maturation in multiple brain structures (Katz and Shatz, 1996; Tritsch et al., 2007; Griguoli and Cherubini, 2017) and develop into adult-like forms with different timetables as sensory capabilities emerge (Fox, 1995; Garaschuk et al., 2000; Lendvai et al., 2000; Jain et al., 2003; Khazipov et al., 2004). In the cortex of mammals, most available data derive from the early postnatal period in the visual and whisker sensory systems (Khazipov and Luhman, 2006; Espinosa and Stryker, 2012; Luhmann et al., 2016), whereas later periods and other systems have been investigated less. Here we explore the functional columnar organization of the somatosensory cortex of the hindlimb (S1HL) in anesthetized rats at an age when the pups begin to walk (P14–P16), and we compare these data to adults.

The somatosensory cortex is topographically organized, and the bulk of structural changes appear to be adult-like by P14. The segregation of the hindlimb and forepaw is complete between P13 and P15 in rats, and it is modulated by sensory-motor feedback as in other somatosensory areas (País-Vieira et al., 2015; Seelke et al., 2012). At some point, endogenous unit firing is gradually replaced by synaptic drive, which relies on the development of axonal trees and finally on the maturation of synapses. There is little data on the functional maturation of intra-columnar and horizontal association pathways, which is required for the fine control of the hind limbs in complex motor tasks that are acquired at later stages (Altman and Sudarshan, 1975).

The postnatal refinement of cortical microcircuits would be expected to entail changes in active pathways along the cortical width, which could be investigated through laminar field potentials (FPs). However, the temporal and frequency features of spontaneous FP patterns and oscillations can rarely be assigned to specific connections due to the variable coactivation of multiple populations and the strong contamination of cortical FPs by volume conduction from distant areas (Herreras, 2016; Torres et al., 2019). Indeed, most of the electrophysiological data available come from either *in vitro* preparations that cannot address the spatiotemporal organization of spontaneous activity, or from *in vivo* experiments that use surface EEG or standard analytical techniques to explore raw FPs (Le Bé and Markram, 2006; Chang et al., 2016), neither of which allow separation and laminar specification of the coactivating pathways. An additional difficulty stems from the rapidly changing electrogenesis in the postnatal period, such as the waves of voltage-dependent channel expression, the initiation of the excitatory synaptic transmission, and the change from a depolarizing to a hyperpolarizing action of inhibitory transmitters at their neuronal targets, all of which are typically associated with the first and second postnatal weeks (Alzheimer et al., 1993; Kang et al., 1996; Rivera et al., 1999; Ben-Ari et al., 2004; Moody and Bosma, 2005). The early repertoire of voltage-dependent intrinsic channels and synaptic receptors supports endogenous forms of FP activity (DuPont et al., 2006; Hanganu et al., 2009), but it is not known whether the same pathways and neuron types generate cortical FPs during the transition to adulthood.

In the present study, we investigated spontaneous intermittent slow-wave activity (SWA) with an emphasis on the sources generating delta waves and their laminar distribution, for which we used high-resolution spatial discrimination techniques that enable segregation of the spatially coherent sources blended in multisource signals such as the FPs (Herreras et al., 2015). To achieve a more complete picture, we also obtained the laminar profiles of unitary firing and those of current-source density (CSD) of evoked responses upon peripheral stimuli. We found highly discrepant laminar profiles between juvenile and adults for both spontaneous and evoked activities. Only some of the laminar sources in juveniles are present in adults, while those dominating SWA in adults, particularly in middle layers, are missing in juveniles. Therefore, SWA is contributed by different sets of synaptic segments during development. The implications for functional sensory-motor control are discussed.

## Materials and Methods

All experiments were performed in accordance with EU (2010/63/UE), Spanish (RD 53/2013), and local (Autonomous Community of Madrid, Order 4/8/1988) regulations regarding the use of laboratory animals, and the experimental protocols were approved by the Research Committee of the Cajal Institute.

### 

#### Experimental procedures and design

Juvenile (P14–P16) and adult (≥P60) Wistar rats were anesthetized with urethane (1.5 g/kg, i.p.), placed in a stereotaxic device, and the body temperature was maintained at 37.5°C (juvenile) or 37°C (adult), using a feedback-controlled heating blanket. We used animals of either sex (P14: 4 males; P15: 4 males, 3 females; P16: 4 males, 2 females; adults: 4 males, 2 females), and the data were pooled because we found no differences in the main results. Juvenile animals were selected from litters that were maintained below six siblings to facilitate body weight control (P14: 35–40 g; P15: 40–45 g; P16: 45–50 g). In this period, the animals had opened eyes, they were able to perform crawling movements and grooming, and some were even at the walking stage. Also, they had an incomplete withdrawal reflex of the limb upon electrical stimulus under anesthesia. In general, juvenile animals showed higher lability to urethane anesthesia than adults. Thus, while adults breathed spontaneously, three series (of 4) of juvenile animals were intubated and ventilated (Midivent 849, Harvard Apparatus) at 120 strokes per minute (heartbeat ∼420/min). In all cases they were given a single dose of atropine (0.01 mg/kg) and dexamethasone (0.4 mg/kg). Glucose-saline was supplemented (10 ml/kg) every hour to maintain the animal hydrated, and experiments never lasted >4 h in total. Surgical and stereotaxic procedures were performed as described previously (Canals et al., 2005). Recordings were obtained with a linear silicon probe (32 sites, 65 or 100 μm inter-site distance) from Atlas Neuroengineering. The short probes were used in experiments seeking high spatial resolution, and mineral oil was used to cover the site of implantation after electrode penetration. The long probes were used to collect voltage profiles spanning the entire cortical width, and the pial surface was covered by ACSF filling a small pool built with a 2 mm wide silicone ring glued to the skull surrounding a bone drill of 1.5 mm. Several recording sites were left in the pool out of the cortex, which allowed visual inspection of the first electrode within the tissue. The built-in reference site was not used to avoid influence from nearby sources that severely distort the spatial landmarks of local FPs. Instead a silver chloride wire implanted under the skin of the neck served as a reference for recordings (note that volume-conducted contributions are readily separated by the independent component analysis (ICA; see next section; Torres et al., 2019). Signals were amplified and acquired using MultiChannel System, hardware and software at a 20 kHz sampling rate. The placement of the probes in cortical area S1HL was stereotaxically guided in adults (in mm): AP: −0.7; L: 3.9. In juvenile animals, the location of the S1HL area was guided by tactile stimulation of the limb to optimize a response over the cortical surface of the contralateral side recorded by micropipette: coordinates were AP: −0.5 to −0.7, and L: 2–2.3. Tactile stimulation was achieved by gentle pushing of the hindlimb skin through a plastic rod attached to a solenoid (0.1-s-long pulses delivered at 0.1–0.2 Hz). Electrical stimulation was achieved through a pair of 35 G needles placed under the skin on both sides of the hindlimb. The intensity was maintained just below the threshold for producing muscle twitch to avoid mechanical artifacts (typically 0.5–1 mA for 0.1 ms pulses). This intensity minimizes nociceptive and muscle responses, and simplifies the cortical activation. For averaging, stimuli were selected when they were given within periods of activity (Up states; Humanes-Valera et al., 2017). In most experiments we stimulated only one limb, either electrically or mechanically, to reduce the time of experimentation. Probes were soaked in DiI before insertion (Invitrogen) to assess their location postmortem in histological sections.

In a series of experiments aiming to selectively and gradually remove the FP generators in upper cortical layers in adults, we blocked AMPAR by delivering a solution of DNQX (Tocris Bioscience) diffusing from the pool at the skull. In pilot experiments we determined that 40–60 min application of 100 μm DNQX at the pial surface was sufficient to completely block the upper FP generators in the cortical tissue surrounding a recording linear probe lowered from the center of the pool. At the end of each experiment the animals were perfused with PB followed by paraformaldehyde (4%) through the abdominal aorta. Coronal brain sections (50 μm) were then stained with bis-benzimide and the electrode position was assessed by fluorescence microscopy. A subset of animals was treated differently for detailed study (see the section Histology).

#### Signal treatment and analysis

Wide-band FPs (0.1 Hz–5 kHz) were recorded in 4 min periods separated by 15 min, and additional high-pass digital filter was set at 0.5 Hz to remove slow transient artifacts. Occasionally, the activity in a single recording site with transient artifacts was interpolated from the surrounding electrodes. Faulty recordings in outer sites were rejected. For quantification, no selection of electrographic state was made for juvenile animals, whereas periods of SWA displaying Up/Down states were chosen in adults (i.e., the activated cortical state was left out of this study). Therefore, although there is no evidence for functional equivalence of the cortices in the two age groups, we sought an electrographic equivalence by selecting FPs that exhibited intermittent periods of activity in all groups. For simplicity we maintained the standard terms Up and Down states to define periods with large delta waves and the intermissions with flat or minimal voltage, respectively, in all age groups. We quantified the mean rate and stability of these periods by building a time envelope of the power with an integration window of 1 s, which was found to be optimal for this purpose. We then used power spectra on these envelopes to obtain a grand total of the average rate and long-term stability of active periods. The energy of FPs was estimated for raw signals as well as for their component frequency bands that were defined to contain the main observed patterns as follows: delta (δ), < 3 Hz; theta (θ), 3–8 Hz; α-β, 8–25 Hz; low gamma (γ), 30–80 Hz. When using the short probes, the inter-animal estimation of FPs was achieved by aligning profiles with respect to the pial surface and using the tracks and DiI marks left by electrodes in histological samples. Because the integrity of histological samples from juveniles was often compromised, in some animals we assessed the electrode localization using electrophysiological criteria, such as the site of polarity reversal for delta waves in Up states, or the characteristic spatial distribution of a pervasive gamma generator (see below). These spatial markers turned out highly stable and matched well histological marks or the visual guide when using the long probes.

##### Spatial discrimination of intracerebral sources by ICA of FPs.

Spontaneous FPs in the brain are contributed by coactivating sources that may be located at different distances from the recording electrodes. Their relative contribution cannot be ascertained in raw FPs. To disentangle these and identify their local or remote origin, we used ICA (Makarov et al., 2010), which belongs to the family of blind source separation methods. The interpretation of ICA-separated components depends largely on the nature and characteristics of the signals (Bell and Sejnowski, 1995; Stone et al., 2002). An ICA is routinely used to elucidate functional connectivity in scalp EEG recordings or in fMRI (Onton et al., 2006), and in recent years also to disentangle source components in intracranial FP recordings (Korovaichuk et al., 2010; Benito et al., 2014; Martín-Vázquez et al., 2018; Munro Krull et al., 2019; Torres et al., 2019). In these recordings, the linear arrays may be placed such that they span the volume occupied by the sources themselves so that the obtained components resolve the electrical fields generated by different afferent pathways even when these make contact in the same neuron population as long as they do not contact identical postsynaptic territories. We thus refer to ICA components as FP sources or generators. We used the kernel density ICA algorithm (Chen, 2006) that was implemented in MATLAB. This algorithm outperforms other more common algorithms (e.g., infomax), particularly for signals with high rhythmic content (Fernández-Ruiz et al., 2013). Recorded FP signals *u_m_*(*t*) are considered as the weighted sum of the activities of *N* neuronal sources or FP-generators:

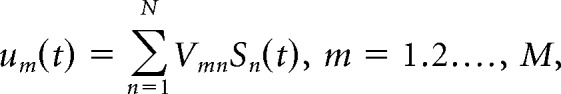
 where (*V_mn_*) is the mixing matrix composed of the so-called voltage loadings or the spatial weights of *N* FP generators on *M* electrodes and *s_n_*(*t*) is the time course of the *n*th FP generator. Thus, the raw FP observed at the *m*th electrode tip is a linear mixture of the electrical activity of several independent FP generators. Using *u_m_*(*t*), the ICA finds both the (*V_mn_*) and *s_n_*(*t*). The joint group of spatial weights (*V_mn_*) is ordered into instant depth profiles of the voltage according to electrode position. In the hippocampus, such curves match the spatial profiles of the standard evoked potentials of specific pathways (Korovaichuk et al., 2010; Benito et al., 2014). In the cortex, this proved less useful to identify the afferent pathway because of sequential activation of several neuron populations whose activities overlap strongly in space and time along the cortical column (Mitzdorf and Singer, 1978). We did not attempt to identify the pathway responsible for each cortical FP generator. Instead we used their spatial landmarks for characterization. In a former study, the overall shape of the spatial distribution was sufficient to identify the local or volume-conducted origin of the contributions to cortical FPs (Torres et al., 2019). Thus, the spatial profiles with maxima within cortical layers belong to local sources, whereas those with linear or quasilinear distribution denote distant sources, either subcortical or from distant cortices.

As for the time course of ICA components *s_n_*(*t*), we earlier showed experimentally and numerically that it reflects the envelope of pathway-specific postsynaptic currents produced by a target population and hence it can be considered a proxy for the temporal convolution of spike output in the upstream population (i.e., afferent spike trains; Korovaichuk et al., 2010; Makarova et al., 2011; Fernández-Ruiz et al., 2012a,b; Martín-Vázquez et al., 2013, 2016). This interpretation is biophysically supported by the instantaneity of electrical fields that makes homogeneous pathways produce electrical fields that are proportional anywhere in space, which is the condition optimized by the ICA. The mathematical validation and practical limitations of the ICA applied to multisite FPs have been thoroughly investigated earlier (for review, see Herreras et al., 2015; for additional considerations relative to cortical FPs, see Torres et al., 2019). Once extracted, each FP generator can be analyzed independently in the time or the frequency domains, or used to reconstruct virtual FPs produced by one or a desired blend of generators (*Ibid*).

In a previous study we found that most of the variance of FPs in the cortex can be accounted for by a few ICA components with distinct spatial distribution (4–7 of a possible 32, a maximum defined by the number of electrodes; Torres et al., 2019). This permits the preprocessing of FPs before performing the ICA by reducing the dimensions through a principal component analysis (PCA), which efficiently diminishes weak noisy generators (Makarova et al., 2011) thereby stabilizing and accelerating the subsequent convergence of the ICA (Makarov et al., 2010). The optimal choice of PCA components is two or three more than those that attained significant variance in the ICA. Although the percentage of contributed variance can be optimized by user-guided selection of recording channels, we routinely disregard ICA components that explain <1% (i.e., always keeping 99% of the original FP variance), unless their spatial and temporal accuracy can be ensured by other means. In adult animals we earlier determined that maintaining the six main PCA components optimizes the ICA's performance in recordings containing mostly SWA in adults. In the juvenile animals, the temporal structure of the signals turned out to be somewhat different, thus the number of PCA components was selected through more cautious scrutiny as has been described previously (Torres et al., 2019), and we used 6–8 PCA components.

##### Current-source density.

One approach to discriminating local contributions to evoked FP profiles involves the use of CSD (Lorente de No, 1947; Mitzdorf, 1985). We used a one-dimensional approach that calculates the CSD as the second spatial derivative of the voltage distribution along the main axis of the cells (Herreras, 1990). For raw FPs we used a differentiation grid equivalent to two recording positions (i.e., 130 or 200 μm), whereas we used 65 μm for evoked potentials. SWA has wide spatial coherence across cortical areas (Destexhe et al., 1999; Torres et al., 2019), which minimizes spurious currents due to unbalanced tangential currents. Although tissue conductivity is rather homogenous across cortical layers (López-Aguado et al., 2001), we do not know the precise values of resistivity in immature brain, which has a much larger volume fraction (Lehmenkühler et al., 1993). Therefore, CSD values are expressed in arbitrary units (V/cm^2^), and we did not attempt to compare the magnitude of currents in juvenile versus adult animals. The short probes allow estimating the CSD over 1755 μm, which is insufficient to cover completely the cortical width. In addition, we detected during the analysis that in some cases the CSD profiles near the pial surface were not as smooth and stable as they were in deeper sites. Consequently, the CSD in upper cortical layers could not be estimated. This problem was alleviated in the experimental series that used 100 μm grid probes and an overlying pool from which the upper 5–6 electrodes recorded from outside the brain. It should be noted, however, that the lower resistivity of the ACSF compared with the brain may have caused a quantitative distortion of the CSD estimated in upper cortical layers.

The cortical layer displaying the earliest evoked response was determined using the CSD instead of the voltage traces, because the large voltage fluctuations during Up states hampered the reliable detection of the initial response in evoked FPs. The latency was taken at the instant when CSD augmented twice over the baseline noise. This procedure also has some drawbacks, such as the uneven spatial offset of evoked currents by spontaneous ones, which remained even after averaging (due to presence of Up states) and may even occlude some weak dipoles. However, we favored the use of CSD as it turned out a more consistent index across the animal population.

In contrast with evoked potentials, in which the baseline before the stimulus can be reasonably assigned to zero, the CSD is not very efficient for spontaneous FP profiles recorded in AC-coupled mode, as they lack a true baseline; hence polarity, phase, and amplitude of resultant CSD waves are unreliable (Brankačk et al., 1993; Martín-Vázquez et al., 2013). To minimize this issue, we offset all recording channels to zero at an instant when synaptic activity is known negligible and no intracortical voltage gradients are expected, i.e., during inactive (Down) periods. An additional cause of spurious currents was the low cutoff filter (0.1 Hz) that produced rebound voltage artifacts at the wake of the large sustained low-frequency waves (Up periods). As a result, spurious currents appeared after Up states during spatial differentiation and we removed them for clarity (except when indicated). Despite the problems that marred the quantitative use of CSD applied to raw FPs (Herreras et al., 2015), it can be used to explore the spatial limits where local currents contribute to spontaneous FPs, which is useful to allocate laminar boundaries in the cortex. These boundaries are less obvious in the spatial profiles obtained by the ICA that take the form of smooth voltage profiles and extend beyond the sources of current (volume-conduction). Thus the CSD and the ICA offer complementary information regarding the spatial and temporal features of the population synaptic activity. However, it should be noted that the CSD is a multisource parameter and the laminar distribution of sources and sinks of current encroach on all coactivated sources in the recorded area (Martín-Vázquez et al., 2013).

#### Spike sorting

For spike detection and sorting, we have used unsupervised software based on superparamagnetic clustering (Chaure et al., 2018), which we found adequate to deal with a possible bias introduced by differences in the biological noise of recordings in juvenile and adults. First, signals were bandpass filtered between 300 Hz and 6 kHz, eliminating the low-frequency band that contains most of the FPs contributed by synchronous activities. The threshold for spike detection was set as *Th* = *N* × median (|*x*|/0.6745), where |*x*| is the absolute value of the signal, 0.6745 is the value for the 75% of the area (*p* = 0.25) of a Gaussian distribution of zero mean and a variance of 1, and *N* is a user defined variable between 3 and 6 (we normally used 5). The resulting thresholds for spike detection were 0.03 ± 0.0003 mV in P14–P16 and 0.022 ± 0.002 mV in adults (values are the median ± median absolute deviation (MAD); W > 6000 and *p* < 0.0001 in all comparisons between adult and juvenile groups, Mann–Whitney–Wilcoxon test), which suggests a slight global under-detection of spikes in the juvenile groups. Concerning spatial regions, the juvenile and adult groups had similar thresholds within the same zone (supragranular: 0.029 ± 0.001 mV vs 0.027 ± 0.006 mV; infragranular: 0.033 ± 0.002 mV vs 0.044 ± 0.015 mV; *p* = 0.6 and *p* = 0.3, respectively, Student *t* test), but we found a significant difference between these two regions (supragranular layer 0.028 ± 0.001 mV; infragranular 0.036 ± 0.004 mV; *p* = 0.04, *t* = −3.4, paired Student *t* test). These values indicated that the count of firing cells was, if anything, biased toward the supragranular layers.

For each detected spike, its wavelet transform was computed, characterizing the spike shapes at different scales and times. In this way, we detected differences in specific features that cannot be distinguished easily in the temporal course. After selecting the wavelet coefficients with higher non-Gaussian variability, a superparamagnetic clustering is performed. Briefly, the algorithm measures the interaction between two points (wavelet coefficients) in function of their separation and a parameter *k*, determining each cluster with the resultant value. Using small *k* values, all spikes are grouped into a single cluster, whereas for higher ones, each waveform is considered independent. Progressively increasing *k*, real clusters are found when a small change in *k* separates a group into two or more clusters with several spikes in each one. We validated the method against a well known supervised method (Quiroga et al., 2004) and confirmed the correct identification of spike clusters.

Units were classified into two subclasses, pyramidal cells (PCs) and putative interneurons (PIs), based on standard electrophysiological criteria that were adapted for the population of juveniles: (1) spike width (>0.3 ms for PCs and <0.3 ms for putative interneurons); (2) mean firing rate (<5 and >5 Hz, and <1 and >1 Hz for PCs and PIs in adults and juveniles, respectively); and (3) the decay of autocorrelograms (fast vs slow).

#### Histology

A subsample of animals (4 juveniles and 2 adults) was chosen for fine histological processing. This served to align the recording sites in relation to cytoarchitectonic areas and layers in conjunction with electrophysiological criteria, and to quantify the width of the cortical layers. Linear probes were soaked in DiI (Sigma-Aldrich) before placement in the tissue. At the end of recordings, animals were deeply anesthetized with urethane and perfused through the left cardiac ventricle with saline and then 4% paraformaldehyde in 0.1 m phosphate buffer. Brains were removed, postfixed in the same solution for 20 h, and cryoprotected in 30% sucrose until they sunk. Brains were then flash-frozen in isopentane (2-methylbutane, Merck), cooled in a 70% ethanol dry ice bath, and stored at −80°C until cutting. Brain tissue was cut into 50-μm-thick coronal slices with a freezing sliding microtome (Microm HM 450, Microm). Sections were washed several times in PB and incubated for 20 min at room temperature in a 0.5% solution of Triton X-100 in PB, followed by 20 min incubation in the green fluorescent Nissl stain NeuroTrace 500/525 (N21408, Life Technologies; ThermoFisher Scientific) diluted (1:200) in PB. After washing, they were then counterstained with the nuclear stain DAPI (4′, 6-diamidino-2-phenylindole; Sigma-Aldrich; diluted 1:80), mounted, coverslipped with ProLong Gold anti-fade reagent (Life Technologies) and studied at 10× magnification by Zeiss 710 microscopy (Carl Zeiss MicroImaging).

DAPI (blue), DiI (red), and NeuroTrace (green) fluorescence from slices were photographed in separate channels, and ZEN 2012 software (Zeiss) was subsequently used to construct composite images from each optical series by combining the images recorded through the different channels. Electrode tracts were reconstructed from the DiI fluorescence. The depth of the limits between cortical layers according to the cell size and density was measured in each section. To estimate the shrinkage in our samples, we measured the surface area and thickness of the sections using Adobe Photoshop and Stereo Investigator (MBF Bioscience) software, respectively, before and after tissue processing for immunofluorescence. The surface area after processing was divided by the value before processing to obtain an area shrinkage factor (p2) of 0.76 in P14, 0.78 in P15 and P16, and 0.84 in adult animals. For correction of lineal measurements in the *x–y* plane we used a volume shrinkage factor (*p* = √*p*2) of 0.87 in P14 and P15, 0.88 in P16 and 0.92 in adult animals. Similarly, we measured the total section thickness at multiple points (>10) of every section before and after tissue processing. After averaging the mean values from all sections, we obtained linear shrinkage factors in the *z*-axis (pZ) of 0.86 in P14, 0.87 in P15, 0.90 in P16 and 0.93 in adults. Corrected values corresponding to the depth of limits between layers and to section thickness were superimposed on the electrode tract reconstructions. Distinction of the boundaries between cortical layers relied on apparent changes in cell morphologies, sizes, and packing densities.

#### Statistical analysis

##### Data collection.

We used 23 rats using the short grid recording probes, 5 for each age group, and 3 additional adult animals using the long grid probes. Some estimations could not be obtained in all animals but we ensured that at least an *n* of at least 4 was available for important measurements in the juvenile to adult comparisons. When there was no statistical difference among P14, P15, and P16 groups, they were pooled together. FPs were recorded simultaneously from 32 recording sites at 20 kHz sampling rate and stored for further offline data analysis. All statistical analyses and data treatment were performed in MATLAB using the Statistics and Machine Learning Toolbox. The ICA was performed using LFPsource software running in the MATLAB environment and freely available at http://www.mat.ucm.es/~vmakarov/downloads.php. For a detailed description and examples, see Herreras et al. (2015).

##### Descriptive statistics.

All data are presented as either the mean ± SEM or the median ± the MAD if the distribution deviated significantly from normal. To check for normality we used the Kolmogorov–Smirnov test. Individual data points and mean values are shown for all graphs in all figures. We used the Statgraphics software for statistics. FP amplitude histograms and skewness were estimated in MATLAB.

##### Hypothesis testing.

We used either one-way ANOVA followed by Bonferroni *post hoc* test or the nonparametric Wilcoxon signed rank test (with 5% level of significance). The effect size *r* for the Wilcoxon signed rank test was computed as *r* = *z*/√*N*, where *z* is the *z*-score value for the T parameter and *N* is the number of samples.

##### Independent component analysis.

ICA is implemented as part of the LFPsource software and uses the fast kernel density method (Chen, 2006) with prior data conditioning: raw FPs (an epoch with 32 channels) undergo PCA and <1% of the data variance containing noisy signals was eliminated. This yields an appropriate dimension reduction. ICA of the reduced dataset was then applied. Grouping of spatial distributions from similar generators was achieved by hierarchical clustering, evaluating the distance measure between curves using the Icasso software package (http://www.cis.hut.fi/jhimberg/icasso/). The dissimilarities among components were computed as the sum of the square differences between two spatial profiles. The function *hcluster.m* was then used to determine each cluster. The sample sizes were similar to those reported in previous publications using ICA for the segregation of FP generators and their quantification (Martín-Vázquez et al., 2013, 2016; Benito et al., 2014, 2016; Torres et al., 2019).

##### Spectral analysis.

The occurrence of oscillatory bouts in specific frequency bands of raw FPs was detected through wavelet spectrograms. The threshold was set to twice the SD of the mean value in the period analyzed. We estimated the power spectral density of the temporal activation of FP generators (periodogram) and then computed the signal power in different frequency bands.

##### Power of FP generators.

The time evolution of the power of an FP-generator (in mV^2^) was calculated by the following:

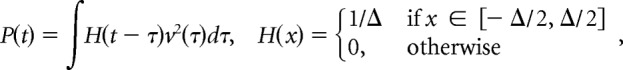
 where *v*(*t*) is the virtual FP at the electrode with maximal power and Δ is the length of averaging. The overall mean power is then defined by setting Δ equal to the complete time interval.

##### Cross-correlation.

Coarse synchronization between different recording sites and time courses of LFP generators was estimated using the cross-correlation coefficient (CC) and spectral coherence. The CC was obtained as follows:

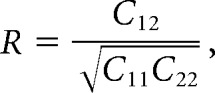
 where (*C_ij_*) is the covariance matrix of two random variables. We used epochs of 60 s for analysis. Spectral coherence was calculated by the following:

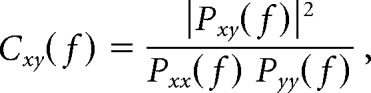
 where [*P_ij_*(*f*)] is the matrix of cross-power spectral density. To determine the level of significance we used the surrogate data test. Randomizing phase relations and keeping other first order characteristics intact, we obtained surrogate time series from the original signals. For each experiment we generated 400 surrogates and we evaluated pairwise spectral coherences. The level of significance (at α = 0.05) was then calculated for each frequency value and coherence above this level was considered statistically significant.

## Results

### Spatial and temporal differences in S1HL cortical field potentials in juveniles and adults

**Figure 1.**
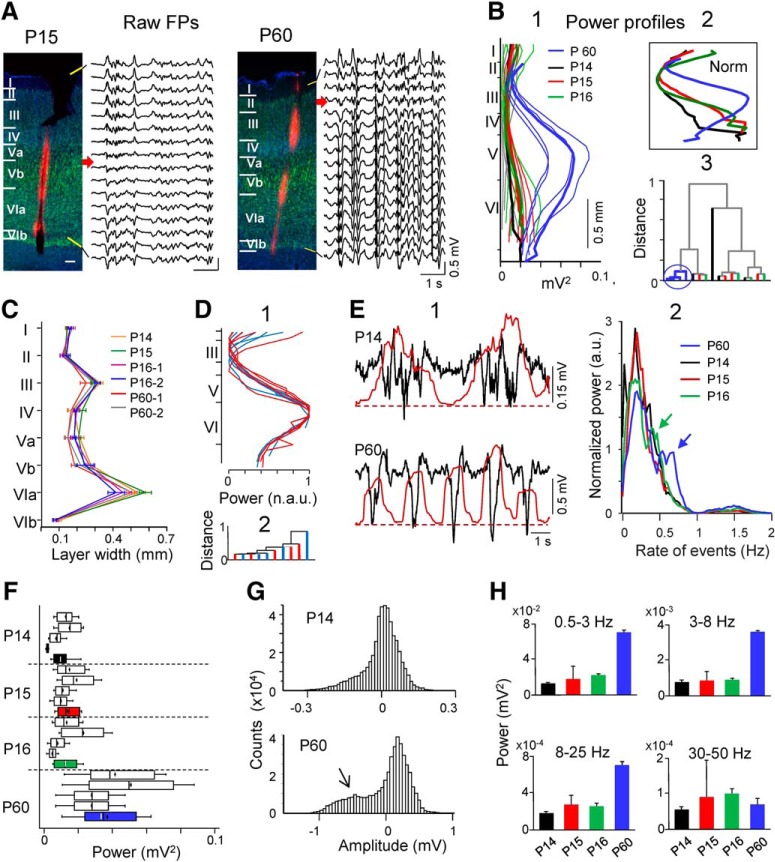
Spatiotemporal differences of cortical FPs in juveniles (P14–P16) and adults (P60). ***A***, Photomicrographs from Nissl fluorescence-labeled sections counterstained with DAPI showing distribution of the DiI-marked probe track through the HLS1 cortex from P14 and P60 rats. Layers (I–VIb) and the depth of the cortical layers (μm) are indicated. Scale bar, 143 μm. To the right, sample FP periods along the cortical width. Arrows point to the site of polarity reversal for the dominant delta activity. ***B1***, Laminar profiles for the mean power activity (V^2^) obtained for all animals (thin traces) and the mean computed for each group of age (thick traces). Profiles were aligned according to histological and electrophysiological criteria. ***B2***, Normalized mean profiles per age group. ***B3***, Clustering analysis of the voltage profiles for the 16 animals: only profiles in adults were clustered in a single class (blue oval), whereas those from juveniles were intermingled, indicating larger spatial diversity. ***C***, Detailed anatomical study of the layer width shows similar values in all age groups. Each point represents the mean ± SEM for the specified layers in each animal. ***D***, Increasing the level of global anesthesia does not produce changes in the shape of the mean V-profile across the S1HL cortex. ***D1***, Normalized mean V-profiles obtained for initial (1.5 g/kg; red traces) and reinforced (2 g/kg; blue traces) urethane anesthesia in juveniles (*n* = 5). ***D2***, Hierarchical clustering shows no age-dependent grouping of V-profiles. ***E1***, The FPs (black traces) recorded in layer V were smoothed as time envelopes of the variance to remove fast fluctuations (1 s time window; red traces), and the mean rate of these episodes appeared in normalized power spectra (***E2***, arrows). ***F***, Integrated mean power across layers I to VI for each animal (open boxes) and the mean (black boxes) of each group of age. The statistics shown are the two middle quartiles (boxes), the median (middle line), the mean (plus sign), and the whiskers are the upper and lower ranges. ***G***, Amplitude histograms of the FP recorded in layer V for two representative experiments. Juveniles show monomodal shape while adults display bimodal distributions (arrow). ***H***, Frequency content of FPs recorded in layer V. Values correspond to the mean and SEM of the power within the indicated frequency band in four animals per group (statistics in the text). All experiments were made with the short probes except in ***D***.

All age groups displayed FPs organized as intermittent periods of activity and silence akin to synchronized cortical electrographic states in adults. These are not uniform and the varying activities in different synaptic pathways lead to layer-specific phenomena. As a first approach to laminar exploration of FPs we built laminar profiles of the mean power (V^2^) using linear probes with high spatial resolution (short probes). The profiles were aligned with respect to the pial surface using histological marks (Fig. 1*A*). All power profiles exhibited a U-shaped distribution with two-maxima within the cortical width caused by the minimum amplitude at the position of the polarity reversal of the dominant delta activity. The voltage profiles were negative in deep layers and positive in superficial ones. There were marked differences among age groups. The polarity reversal in adult animals was rather abrupt at ∼ 400 μm below the pial surface (layers II–III), whereas it was shallower and in some cases barely apparent in juveniles, and was centered 350–500 μm deeper compared with adults (layers IV–V; Fig. 1*A*, red arrows). The power profiles were better defined at P16 compared with P14 (Fig. 1*B1*), and we used hierarchical clustering of all normalized profiles to explore similarities (Fig. 1*B3*). Only those from the adult group were clustered (blue oval), whereas those in P14–P16 were intermingled, suggesting a similar and more stable composition and laminar zonation of dominant pathways contributing to FPs in adults. An age-dependent shift of the maximum power toward upper layers is appreciated in the normalized averaged profiles (Fig. 1*B2*), which was located in the middle of layer V in adults, whereas it was in layer VI in the P16 group. P14 and P15 groups did not display a clear maximum within the cortical limits.

We explored the possibility that the different voltage profiles in juveniles and adults was caused by structural differences in the gross cytoarchitecture of the cortex. Thus we measured the width of cortical layers in histological samples of the S1HL area in two adults and four juvenile individuals (Fig. 1*C*). Cortical layers were identified as layers I, II, III, IV, Va, Vb, and VI, Zilles and Wree (1995) and Fiáth et al. (2016). We found no significant difference between adult and juvenile cortical layer thickness (*p* > 0.1 in all cases, Mann–Whitney–Wilcoxon test), indicating that at least the lamination and general distribution of cells within the S1HL area has already achieved an adult configuration by P14–P16. These observations point to microstructure and/or functional factors as the origin for the age-dependent laminar differences in S1HL FPs.

Among the latter factors, it is known that different levels of anesthesia have a marked effect in the temporal pattern of cortical FPs, which might translate on differences on the V-profiles as well. Thus we explored their sensitivity to anesthesia levels in juvenile animals by increasing the initial dose of urethane from 1.5 to 2.0 g/kg, and we used the long probes to span the entire cortical width. As reported for adults, the mean power estimated over 60 s periods in a group of five P15 animals reduced to 73% of the value at the initial dose (0.042 ± 0.02 vs 0.033 ± 0.02 mV^2^). However, the power profiles obtained at the different anesthetic doses exhibited the same laminar distribution (Fig. 1*D1*; profiles were normalized to account for the variation in power), and the hierarchical clustering did not reveal any age-dependent grouping (Fig. 1*D2*). We should mention that despite a reduced rate of Up states at the high anesthetic dose, the overall electrographic state remained. Throughout all experiments, the comparisons of S1HL FP activity between juveniles and adults were made with identical anesthetic level and homologous electrographic state.

We also characterized the temporal and frequency signatures of cortical FPs to gain a wider spatiotemporal understanding of cortical FP dynamics. For simplicity we use the widely used terms “Up/Down states” in both age groups. In adults, this pattern constitutes the customary SWA composed of large delta waves lasting 0.4–0.6 s and recurring at ∼0.5–5 events per second (mean 0.34 ± 0.06 events/s; Fig. 1*E*; see Materials and Methods for customized rate estimation). Up states appeared as intermittent spatially coherent homogeneous waves with smooth voltage gradients over the cortical width, or the waves can be heterogeneous, displaying several negative deflections in different laminae that were interpreted as coalesced delta waves of shorter duration. Up states in juveniles showed multiform activity (Fig. 1*E1*, top trace), but they often appeared as series of 4–5 delta waves of short duration (0.1–0.2 s), decreasing amplitude, and a simple dipolar laminar profile (see Fig. 3*A*). Bouts of gamma oscillations appeared associated to some delta waves in all age groups. These active epochs recurred at a slower rate than in adults (0.22 ± 0.02 events/s; mean of 12 animals, 4 per age group). Only the adult group showed significant rate differences when compared with all other groups of age (*p* < 0.01, one-way ANOVA and Bonferroni *post hoc* test). In our animal sample we did not observe spindle oscillations that characterize earlier postnatal periods (Yang et al., 2009; An et al., 2014).

Multiple comparisons yielded the following main observations. The mean power estimated as an average across the cortical layers (I–VI) was 0.011 ± 0.0004 and 0.04 ± 0.003 mV^2^ in juvenile (P14–P16 data were pooled: *n* = 12) and adult animals, respectively (mean ± SEM of 3 observations per animal, 60 s each one-way ANOVA and Bonferroni *post hoc* test, *p* < 0.001; Fig. 1*F*). We found no statistical difference between juvenile groups. Because the mean power may be influenced by the notable instability in the temporal features of the Up states (Bernardi et al., 2018), we also examined amplitude histograms of the FPs obtained in layer V. Whereas in adults these showed a bimodal distribution reflecting the two dominant amplitudes in Up and Down states (mean amplitude: 0.89 ± 0.1 mV, *n* = 15 observations in 5 animals), the juveniles displayed a single mode, denoting the lack of structured bistable voltage fluctuation (Fig. 1*G*). We found no statistical difference in the skewness of amplitude distributions over the P14–P16 period (P14: −0.77 ± 0.12; P15: −0.87 ± 0.14; P16: −0.75 ± 0.07, *n* = 5 in all groups; *p* = 0.84, *F* = 0.28, ANOVA), although some individuals did show an incipient temporal structure evolving toward the bistable pattern of amplitude.

We then analyzed the frequency content of the raw FPs, which turned out markedly layer dependent. However, because the interpretation of the frequency signatures at a single site is uncertain due to extensive volume conduction between layers as well as from remote cortical and extra-cortical sources (Torres et al., 2019) we chose to present only the results obtained in layer V to illustrate the gross differences between age groups (Fig. 1*H*). All frequency bands showed an increasing trend in the mean power with postnatal age that was nonsignificant overall, although some pairwise comparisons were significant, such as between P14 and P16 groups for the delta band (0.5–3 Hz: *p* = 0.005; 3–8 Hz: *p* = 0.7; 8–25 Hz: *p* = 0.08; 30–50 Hz: *p* = 0.05, one-way ANOVA and Bonferroni *post hoc* test). In addition, all but the low-gamma band (Student's *t* test, *p* = 0.4) exhibited a much reduced power when compared with adults (*p* < 0.001 in all cases). However, we did find some differences in gamma oscillations between juveniles and adults. The mean frequency measured in upper layers was 42.3 ± 1.4 Hz in adults, whereas it was 35.7 ± 3.1 Hz in juveniles (data from 62 observations in *n* = 5 and 15 animals, respectively; W = 1740.5, *p* = 0.02, Mann–Whitney test).

### Delta-associated current sinks are reduced in the supragranular zone in juveniles

**Figure 2.**
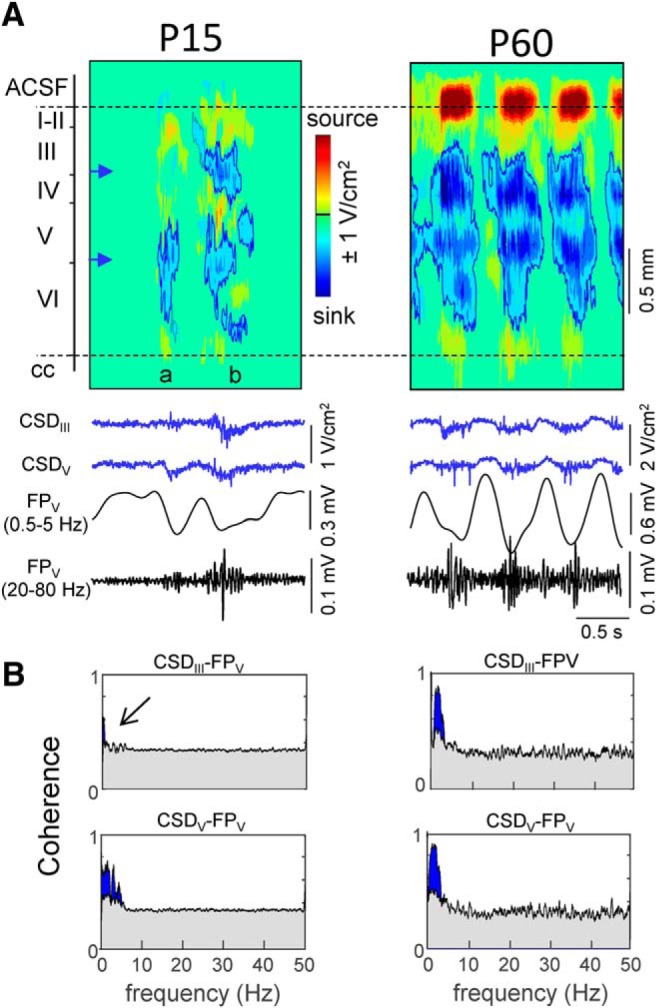
Delta associated sinks of current are not developed in supragranular layers of juveniles. ***A***, CSD contour plots obtained for 2 s samples across the S1HL width in a juvenile (left column) and an adult (right column) using long probes inserted through an ACSF-filled pool at the pial surface. Horizontal lines mark the cortical boundaries. Standard delta waves showed sinks only in deep layers (type a), and some displayed sinks in upper layers when combined with gamma oscillations (type b). The arrows mark the positions where delta-associated sinks of current were stronger in layers III and V, and the temporal traces (blue) were used for quantitative analysis. The black traces correspond to the FPs in layer V filtered in the delta and gamma bands. ***B***, spectral coherence between CSD and FP at specified positions. Blue and gray areas mark the significant and no significant correlations, respectively (Δ*t* = 60 s; surrogate test, *n* = 400). Significant coherence was found in all cases except between FPs in layer V and CSDs in layer III in juveniles (arrow).

**Figure 3.**
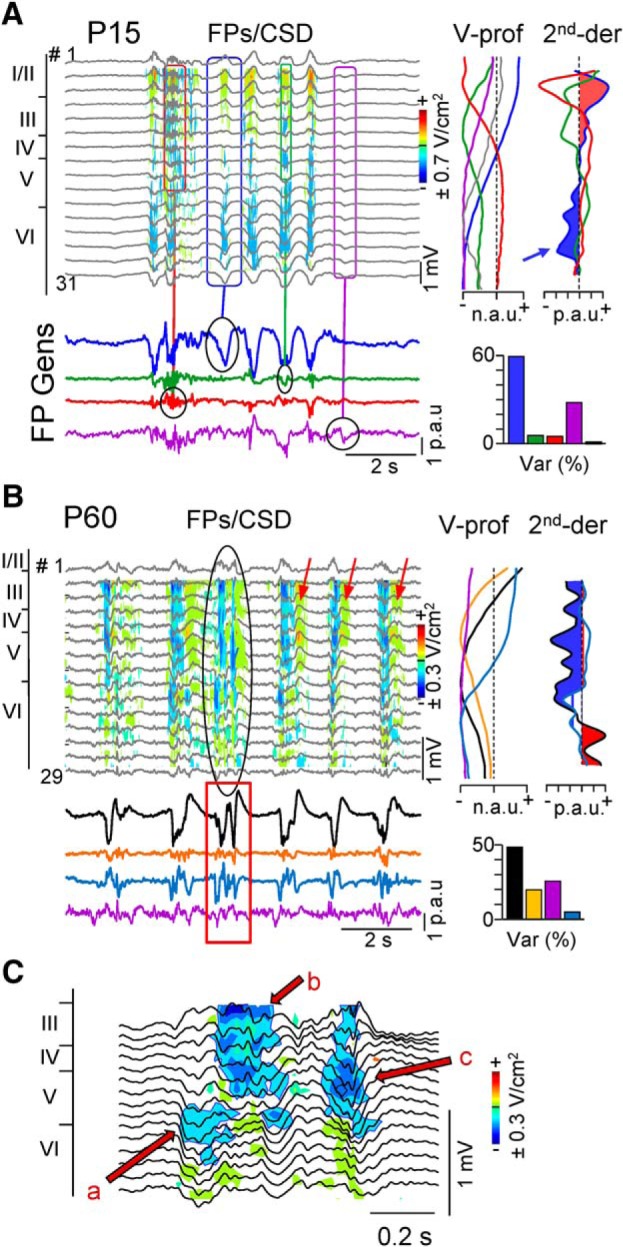
Spatial analysis shows different sets of FP generators in juveniles and adults. ***A***, ***B***, ICA analysis for FPs in two representative juvenile (P14) and adult animals (P60). The top traces correspond to raw FPs (only every other site is shown) and the colored traces below are the time courses of the main ICA-separated FP generators (color coding is maintained throughout all figures). The boxes and circles mark the different spatial coverage of sample FP waves, whose activity is selectively segregated into different FP generators. Superimposed on FPs are the spatiotemporal groups of current sources and sinks obtained by CSD (yellow/red and blue, respectively; near 0 values have been blanked for clarity). Spurious currents were produced by AC-filtering at the wake of Up states (***B***, red arrows), which have been removed in all other CSD maps. The voltage profiles of found FP generators and their second spatial derivatives (CSD-like plots for specific generators) are shown to the right. Those corresponding to the FP generators of interest are filled (color code is interpreted as for CSD). The arrow points to the sink in deep layers for the main generator (blue trace). The histograms show the relative variance of separated FP generators (10 min samples). Note that the dominant generator in juveniles (blue) associates to sinks of current in layers V/VI, whereas the dominant generator in adults (black) associates to broad sinks in middle/upper layers. In the latter age group, a small FP generator (cyan) is revealed that shows similar activity and spatial profile to the dominant one in juveniles (blue). ***C***, Enlargement of the complex delta wave marked in ***B*** in the adult animal. Note the multiple foci of sinks of current (red arrows) spanning middle layers. Each sink associates to negative FP strokes spanning different groups of electrodes along the cortical width, and separated into different FP generators (***B***, black and blue FP_Gens_ in red box). Normalized arbitrary units (n.a.u.) and proportional arbitrary units (p.a.u.) optimize spatial waveform detail and relative power, respectively.

We first investigated the sites where neuronal currents build up the FPs by estimating the CSD of raw FP profiles using long probes that spanned from an ACSF pool above the pia down to the corpus callosum. Most delta waves in juveniles exhibited uncomplicated dipoles of current in which the sink spanned layer VI and the lower half of layer V, and a weak source extended toward the supragranular zone (Fig. 2*A*, sample wave *a* in contour plot). This pattern was observed in all juveniles regardless of the age. Sinks of current were not observed associated to delta waves in middle and upper cortical layers except in cases when these were associated to bouts of gamma oscillations (e.g., wave labeled *b*). The CSD profiles of delta waves were markedly different in adults (Fig. 2*A*, right column), which showed broad sinks with multiple spatiotemporal foci that globally spanned layers III–VI (Fig. 3*C*), whether they were associated to gamma oscillations or not. We quantified these laminar associations in several ways. First, we estimated in juveniles and adults the cumulated sinks of current (sources were removed) over 60-s-long periods in the two layers where they normally peaked in adults, i.e., layers III and V. These were (in proportional units per second) 782 ± 175 and 1143 ± 190 in layers III and V in juveniles (*n* = 6 animals; *t* = 4.02, *p* = 0.01, Student's *t* test), and 1254 ± 224 and 1068 ± 124 in adults (*n* = 7; *t* = −1.03, *p* = 0.34). Next, we cross-correlated the FPs in layer V in which delta waves are present in both groups of age to the time course of CSD estimated in layers III and V (Fig. 2*A*, blue traces), which were 0.42 ± 0.1 and 0.83 ± 0.04, respectively (mean ± SEM of *n* = 5 animals per group, 60 s each, *p* = 0.01, ANOVA). Finally, we examined the spectral coherence for the same comparisons (Fig. 2*B*), which turned out statistically significant for the delta band in all cases except in the comparison between FPs in layer V and CSDs in layer III (surrogate test, *n* = 400; identical results were obtained in *n* = 6 juveniles and *n* = 5 adults). These results indicate a reduced amount of sinks of current associated to delta waves in the supragranular zone of juveniles. Further disclosing of delta and gamma contributions is not possible through CSD, because the currents elicited by coactivated pathways offset each other and the independent contributions cannot be separated (Martin-Vázquez et al., 2013). Thus, we used the ICA to clarify further the age-dependent laminar differences.

### FP generators in middle cortical layers of S1HL are missing in juveniles

**Figure 4.**
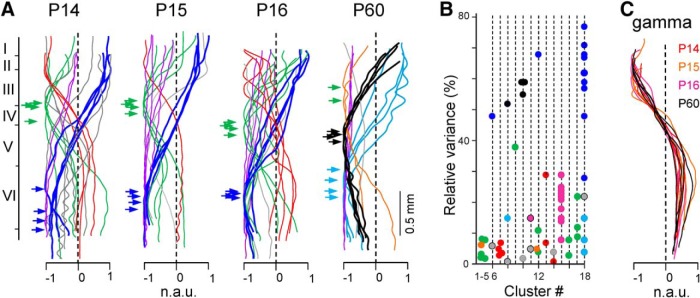
Only some of the FP generators in juveniles persist with similar laminar distribution in adulthood. ***A***, Superimposed spatial distribution of the main generators from all animals and age groups (*n* = 16 animals). Traces in the same color and age group have similar spatial distribution, as estimated by cluster analysis. Arrows mark the site of maxima. The purple traces belong to volume-conducted generators (originated in sites remote to the recording). Some singular generators are depicted in gray. ***B***, Relative variance of all FP generators separated by clusters. Note the grouping into a single class of the dominant generators in juveniles (blue dots) and a weak adult generator (Cluster 18, cyan). ***C***, Superposition of the main gamma generator found in all animals after bandpass filtering (20–80 Hz) to increase the relative contribution of these network oscillations and facilitate their ICA separation. This gamma generator held a remarkable stability with identical spatial distribution in juveniles and adults.

The ICA separates the spatially coherent FPs generated by the different synaptic sources across the S1HL layers, and facilitates exploring the spatial and temporal relations between them (Makarova et al., 2011; Herreras et al., 2015). The presence of multiple synaptic contributions in different laminae is patent in a close-up of FP profiles, in which it can be observed that not all individual FP waves reversed polarity at the same depth (Fig. 3*A*). Some waves exhibited strong voltage gradients along the cortical width and may or may not display a polarity reversal (e.g., blue, red, and green boxes), while others exhibited quasi-linear profiles (e.g., purple box). Such diversity of instantaneous voltage profiles results from variable coactivation of multiple pathways along the cortical width, whose specific profiles were obtained by the ICA. The ICA separated components or FP generators for sample periods in P14 and adult animals (colored traces and V-profiles) are shown in Figures 3, *A* and *B*. The simpler spatiotemporal structure of FPs in juveniles facilitated visual assessment of how waves with identical spatial profiles segregated into the same FP generator (Fig. 3*A*, sample boxed fragments). In each animal, the analysis yielded a set of FP generators that exhibited either a curved V-profile with local maxima within the S1HL or a non-zero quasilinear distribution (purple traces). According to the volume conductor theory, and as also supported by experiments and realistic modeling of multisource FPs (Martín-Vázquez et al., 2016; Torres et al., 2019), the curved profiles corresponded to activity originated in the recorded cortical column (i.e., the S1HL) while the flat ones belong to volume-conducted currents generated at distant sites. We found a main generator in all animals regardless of the age (Figs. 3*A*,*B*, blue and black traces in juveniles an adults, respectively) that explained most of the variance (50–80%), and that exhibited activity that matched delta waves in raw FPs, although the spatial distribution diverged between P14–P16 and adults (Fig. 4 for population analysis). In juveniles, the main generator showed a smooth dipolar profile with maxima in layer VI (negative) and II (positive) and a polarity reversal between layers IV and V (Fig. 3*A*, blue traces). By contrast, the main generator in adults showed a broader, mainly negative potential throughout most of the cortex that peaked in layer V and had a polarity reversal between layers II and III. The maxima in juveniles and adults differed by ∼700 μm. We also estimated the second spatial derivative of FP generators, which provides the mean CSD profile generated by a single synaptic pathway (or a coherent group of pathways: Herreras et al., 2015). Notably, those for the dominant generators (Figs. 3*A*,*B*, right-most solid and shaded plots) roughly matched the CSD profiles obtained from the raw FPs (compare Figs. 3*A*,*B*, 2, CSD contours), which indicated that the bulk of FPs were generated by these dominant generators in both age groups. All other generators in both age groups (except that volume-conducted: purple traces) also produced activity during Up states (Fig. 3*A*,*B*, bottom colored traces), although delta waves were only observed in another weak generator in adults (cyan trace). Note that the coalesced delta waves defining some Up states in adults [Fig. 3*B*, boxed (FPs and CSDs), enlarged in *C*] were separated in two different generators (black and cyan traces). Notably, the V-profile and second spatial derivative of this weak generator in adults were similar to the dominant generator in juveniles (Fig. 3 compare *A*, *B*). Therefore, some generators appear to coincide in both age groups, and we explored this further.

We used hierarchical clustering of the spatial profiles obtained in all animals to search for possible matches between the two age groups, and we targeted the more stable FP generators by analyzing long periods (typically 12 min). Figure 4*A* shows the spatial curves for the main FP generators obtained in four animals for each age group using the short probes. In general, the younger animals returned a higher number of significant FP generators and with higher spatial variability than adults (4.6 ± 0.2 vs 3.5 ± 0.3, *n* = 12 and *n* = 4, respectively). As expected from the diverging maxima, the main generators in juveniles and adults did not cluster together (Fig. 4*B*, Clusters 10 and 18, black and blue dots, respectively). However, the weaker FP generator that peaked in layer VI in adults (Figs. 3*B*, 4*A*, cyan traces) clustered with the main FP generator in juveniles (Fig. 4*B*, Cluster 18, cyan and blue dots), raising the possibility that they belong to the same pathway. In general, juvenile individuals showed a number of weak generators with maxima within the S1HL supragranular layers (Clusters 1–5, 7, 12, 16, 17, green and red dots), whereas these were infrequent in adults (Clusters 5 and 12, brown traces and dots). Along with the heterogeneous sinks in middle cortical layers in adults, these observations suggest that in this age group, the main (black) generator is a multipart one that cannot be disentangled during Up states because of excessive temporal coherence (Makarova et al., 2011; but it may during cortical activated states: Torres et al., 2019), whereas in juveniles it is missing, or its components are incipient and disaggregated.

Some activity patterns, such as gamma oscillations, barely contribute to the total variance of the FP because they appear only occasionally, have short duration and low voltage, and therefore cannot be well resolved by applying the ICA to raw FPs. However, their relative contribution can be augmented by bandpass filtering (20–80 Hz) and the ICA returns the spatial profiles accurately. In this study, we paid attention to the strongest gamma generator that is responsible for most of the variance in the supragranular zone, and we found that all animals displayed nearly identical profiles, regardless of the age group (Fig. 4*C*). Gamma-associated currents appeared throughout most of the cortical width (Fig. 2, wave labeled *b*). Indeed, the bouts of gamma oscillations presented similar spatial structure and constituted the most stable activity in juveniles and adults.

### The blockade of excitatory transmission in upper and middle cortical layers in adults renders juvenile-like FP profiles

**Figure 5.**
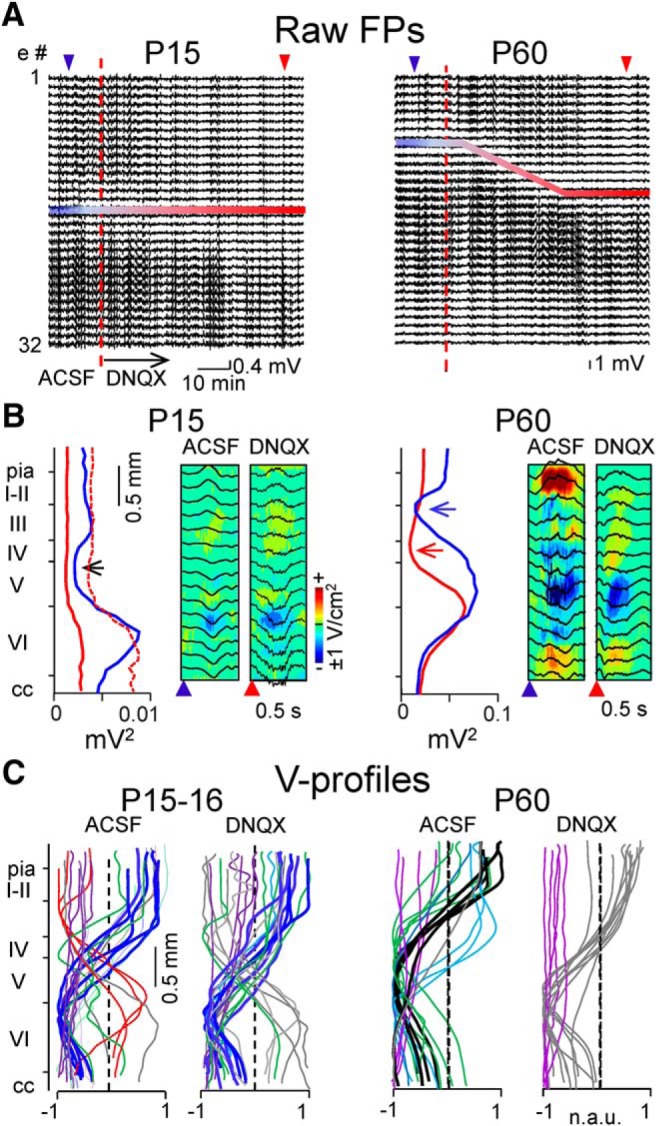
Blockade of excitatory synaptic currents in upper/middle layers in adults recovers juvenile-like FP profiles. Representative animals (***A***, ***B***) and population data (***C***) are shown for the juvenile and adult groups (left and right columns, respectively). DNQX was applied to the pia from a pool built at the skull. ***A***, Compact display of the FPs recorded with a long probe for 80 min before (ACSF) and after DNQX was applied at the moment indicated by the red dashed line to block excitatory transmission in upper/middle layers. A downward shift of the low-power layers reflecting the polarity reversal of dominant delta activity was observed in adults, but not so in juveniles that only exhibited an overall reduction of power (blue-red bars). ***B***, Spatial profiles of the mean power obtained for 5 min periods in control (blue) and after stabilization following DNQX (red). The arrows mark the site of polarity reversal for delta waves, which shifted downward in adults but not in juveniles, as noted in the normalized trace (dashed line). The spatiotemporal CSD contour maps for illustrative delta waves show little effect on the distribution of current sinks in juveniles and the selective disappearance of these in supragranular zones in adults after DNQX. ***C***, Main FP generators obtained in *n* = 5 experiments. Note the disappearance of the dominant FP generators with maxima in layer V in adults (black traces), which were replaced by others with maxima in layer VI (V-profiles, gray). Meanwhile, the dominant FP generators with maxima in layer VI in juveniles (dark blue traces) remained after DNQX. Volume-conducted FP generators (purple traces) that originated in remote sites were not affected by local blockade of excitation.

These findings indicate that multiple generators coactivate during delta waves in adults, and one of them that was weak in this age group, was already present and dominated FPs in juveniles. The inference is that strong currents develop in middle layers in adults to shape the broad main FP generator. We sought additional evidence by removing the synaptic inputs to upper and middle cortical layers through the blockade of excitatory transmission in juveniles (Fig. 5*A*) and adults (*B*). For this purpose, we delivered the AMPAR blocker DNQX (0.1 mm) from a pool at the pial surface while recording through the cortical width using a long probe. Compacted displays of FPs in sample experiments (80 min) showed the gradual transformation of FPs in adults such that the main polarity reversal shifted within ∼40 min to a site 600 μm deeper around the border between layers IV and V. In juveniles the effect of DNQX affected mostly to the overall power, but did not alter the reversal site, which was already located at a deeper site in control (Figs. 5*A*). The mean power profiles obtained over 5 min periods in control and after stabilization reflected the shift in adults as a translocation of the site of minima (Fig. 5*B*, arrows), and a strong reduction of FPs from pia through layer V. Note that FPs do not disappear completely in the upper layers bathed by DNQX, which indicates that they belong to passive currents from excitatory inputs in lower layers. This can be appreciated in the CSD profiles of Up states, which lost the broad sinks in middle layers and became restricted to layers V–VI (Fig. 5*B*, P60, right panels), much alike the spatial pattern in juveniles in which passive outward currents are clearly visible in upper layers in control and under DNQX (Fig. 5*B*, P15, right panels). The population data containing the main FP generators obtained in five experiments for each age group before (ACSF) and after blockade of excitatory transmission in upper/middle layers (DNQX) are shown in Figure 5*C* (P15–16). In adults, the dominant FP generators (black traces) disappeared, along with other generators in supragranular layers (green traces), whereas the volume-conducted (purple) and layer VI generators remained (Fig. 5*C*, P60, cyan and light blue). However, in juveniles only the FP generators with maxima in upper layers disappeared (e.g., the pervasive gamma generator), whereas the dominant generators in lower layers remained. It can be appreciated that the surviving generators in adults showed spatial profiles tightly matching the dominant generators in juveniles.

### Volume-conducted potentials contribute similarly to cortical FPs in juveniles and adults

Earlier we found that currents generated in other cortices or in subcortical regions contribute notably to FPs through volume conduction, and this contribution presents a non-zero linear laminar profile (Torres et al., 2019). In this study, a quasilinear volume-conducted generator also appeared in S1HL recordings in all animals (purple traces), which then must have originated in a region bearing activity that was non-coherent with the S1HL. We observed a marked power decay in this linear generator from mid to upper cortical layers in juveniles (Figs. 3*A*, 4*A*, purple traces), whereas it was almost flat in adults (Figs. 3*B*, 4*A*). Although in principle this provides the juvenile and adult linear generators with their own identity, we showed earlier that the power decay of a remote FP generator along the cortical width may be more or less pronounced depending on the size of the remote sources and the distance to recording (Torres et al., 2019). In this regard, we observed an age-dependent overall trend toward increasing equalization of the power of this generator across the cortical width (Fig. 4*A*, purple traces).

To gain insight into the possible error introduced by volume-conducted potentials in quantifying local FPs in different layers of juveniles compared with adults, we reconstructed the FPs in infra and supragranular layers from ICA components (so-called virtual FPs) leaving out the contribution from the linear ones (volume-conducted contamination). Only the infragranular layers showed significant mean power difference in juveniles compared with adults (0.012 ± 0.001 vs 0.034 ± 0.06 mV^2^, respectively; *t* = −5.7, *p* = 10×^−6^, Student's *t* test, *n* = 36 observations in 12 postnatal animals and *n* = 12 observations in 4 adults).

### Temporal correlation between FP generators in juveniles and adults

**Figure 6.**
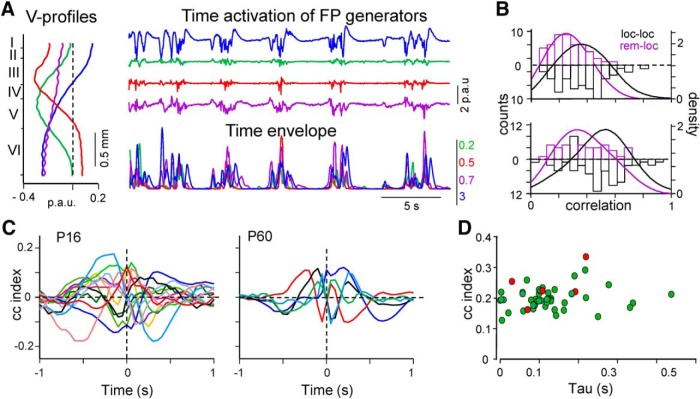
Approximately half of pairs of FP generators held temporal correlation. ***A***, Top colored traces correspond to the time activation of four different generators in a sample epoch for a representative animal, whose spatial voltage profiles are depicted to the left. Bottom traces correspond to the time envelopes for each generator (time window: 0.2 s). ***B***, Pairwise linear correlations of time envelopes across the entire population of animals. Pairs were classified into two groups that either contained the remote generator (rem-loc) or did not (loc-loc). Top and bottom show the same analysis for time envelopes built with 0.2 and 1 s windows, respectively. Bottom correlation was found when the remote generator is included in the tested pairs. ***C***, Superimposed plots of the significant cross-correlation functions found between pairs of generators in animal groups P16 and adult (surrogate test). ***D***, Lag differences (tau) between significant pairwise correlations in ***C*** for the entire population. Green and red circles represent juvenile and adult correlations.

The time course of FP generators reflects the envelope of activity in a pathway, i.e., a cortical segment in the case of intra-columnar connections. Because the ICA preserves the full time resolution of the local as well as the remote contributions to the FPs recorded across a single track (Torres et al., 2019), we looked for possible temporal relations between the ICA-separated FP generators in all age groups. We first estimated the pairwise linear correlation between the temporal envelopes of FP generators (Pearson coefficients; Fig. 6*A*) and established two groups for comparison, those that contained the remote component (inter-areal group) and those that did not (intra-columnar group). Despite the marked Up/Down temporal structure of all FP generators (Fig. 6*A*) that promoted high pairwise correlation values, the results indicated a significantly lower correlation in the group that contained the remote generator: 0.38 ± 0.02 versus 0.5 ± 0.02 and 0.27 ± 0.02 versus 0.37 ± 0.02 for correlation time windows of 1 and 0.2 s, respectively (*t* = −3.4, *p* = 9 × 10^−4^, and *t* = −3.8, *p* = 2 × 10^−4^; data from 59 and 49 pairwise correlations in *n* = 16 animals). The data are thus compatible with a higher functional relation between true local S1HL generators than that observed between S1HL and other areas (implicit in the remote FP generators). This can be interpreted as an expected increased correlation between local network segments in a cortical column compared with remote activities that reached the recording site from remote cortices or other structures. We did not find any significant difference in the comparisons between juveniles and adults (CC: *p* = 0.35, *t* = −0.97; τ_max_: *p* = 0.12, *t* = 1.66, Student's *t* test).

We further explored the temporal correlations between FP generators in each animal through pairwise CCs. Each was obtained as a mean of partial CCs obtained for 30 s periods over long periods (up to 12 min per animal; time step of 10 s), and we used a surrogate test (2000 replications, *p* < 0.05) to find significant correlations. We found 41 of 101 CCs significant in juveniles and 5 of 18 in the case of adults (data from 12 and 4 animals, respectively; Figs. 6*C*,*D*). Some individuals had no significant correlations among their FP generators (2 of 16 juveniles and 2 of 4 adults). The mean delay (τ_max_) of the significant correlations was = 149 ± 20 and 99 ± 26 ms in juveniles and adults, respectively (not significant, *t* = 0.98, *p* = 0.33, Student's *t* test). It should be noted that the two age groups are not comparable as they included different collections of pairs. As indicated, the ICA could not separate the individual components contained in the broad generator during Up states in adults, most likely because of the high coherence between them.

### Distinct laminar distribution of S1HL evoked potentials in juveniles and adults

**Figure 7.**
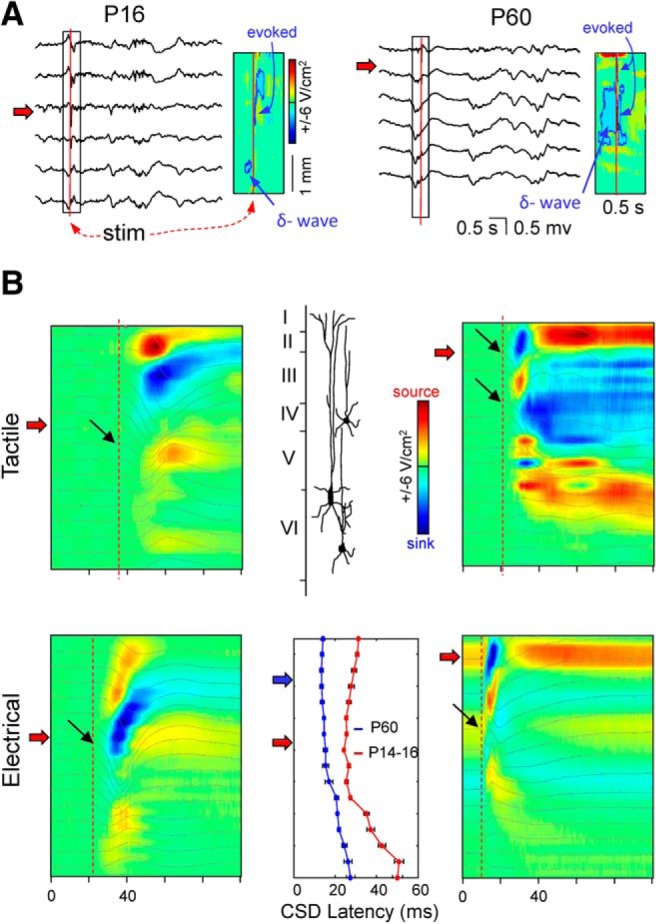
Laminar profiles of FPs evoked by peripheral stimuli differ in juveniles and adults. ***A***, Sample FPs (1 every other 3) for juvenile (left) and adults (right) during spontaneous activity and peripherally evoked responses. The instants of electrical stimuli during delta waves are marked by vertical red lines, and the estimated currents for the boxed fragment are shown to the right (sinks were outlined for visibility). Note that the evoked currents coincided with those associated to delta waves in some but not all layers, which occluded partially the fast-evoked sinks, particularly in layer IV (black arrows). The horizontal arrows mark the site of polarity reversal for delta waves. ***B***, Averaged responses for FPs and CSDs (*n* = 15) selected during Up states. The vertical dashed lines mark the time of the earliest response in the FPs (stimuli are at 0 time), and the black arrows point to the layer where it appeared, typically in or close to layer IV. This may differ somewhat in CSDs because of uneven spatial offset with ongoing delta waves. Note opposite polarity of apparent dipoles at similar locations in juveniles and adults. Bottom, Middle, The mean latency of the earliest averaged CSD at each layer in the juvenile and adult groups upon electrical stimuli in the hindlimb (mean ± SEM). Note the much longer latency in juveniles than in adults.

From the above results, we inferred that the synaptic pathways making up spontaneous FPs in the S1HL cortex do not have the same relative contribution or temporal pattern, and more importantly, do not have the same postsynaptic territory in P14–P16 individuals compared with adults. Because spontaneous activity may engage different networks over time, we explored the laminar distribution of evoked FPs following natural (tactile) and electric (subcutaneous low intensity) stimuli in the hindlimb (see Materials and Methods), averaged during periods of activity (Rigas and Castro-Alamancos, 2009). Forelimb stimuli of either type did not elicit any consistent FP in the S1HL in adults (3 cases), and only did so in one (P14) of four juveniles tested when electrical stimuli was used. Contralateral stimuli evoked FPs that were dramatically different in P14–P16 compared with adults. Regardless of the stimulus type, evoked FPs in juvenile animals were simpler and more variable, had much longer latency than adults and, contrary to the adult group, an epoch of activity (Up state) was not elicited (Fig. 7).

A close look at the laminar distribution of the FPs and the underlying CSD distribution revealed major differences depending on stimulus type and age group. In juveniles, either type of stimuli elicited an apparently single negative wave spanning layers III–V and lasting 20–30 ms. By contrast, a shorter (∼10 ms) negative wave spanning layers II–IV dominated evoked FPs in adults and was followed by a broad multiphasic negativity during tactile but not during electrical stimuli. Despite the simplicity of FP profiles in juveniles, they returned several sink-source groups that were partially displaced in time and space, indicating sequential activation of intracortical segments. The earliest CSD group spanned layers II–III (sink in layer III) and the second, layers III–V. The earliest negative wave in adults returned a main dipole in layers II–III, although it has the opposite orientation compared with that found in juveniles (i.e., the main sink was in layer II). These spatial patterns repeated in different animals with some differences, which would be expected from variable temporal overlap among individuals. We also noticed the uneven spatial offset of evoked currents by the spontaneous ones associated to delta waves in Up states (the laminar profiles of spontaneous and evoked currents differ). This may have altered the relative size of evoked currents, and also the precise location of the earliest event. This was at 40.4 ± 2.4 ms in juveniles and 35.9 ± 1.1 ms in adults for tactile stimuli, whereas it was 26.2 ± 0.9 and 14.4 ± 0.6 ms for electrical stimuli (*n* = 180 and *n* = 60 observations from 12 juveniles and 4 adults; W = 2672, *p* = 0 for electrical stimuli and W = 4430, *p* = 5 × 10^−5^ for natural stimuli, Mann–Whitney–Wilcoxon).

### Age and cell-type-specific differences of spike activity

**Figure 8.**
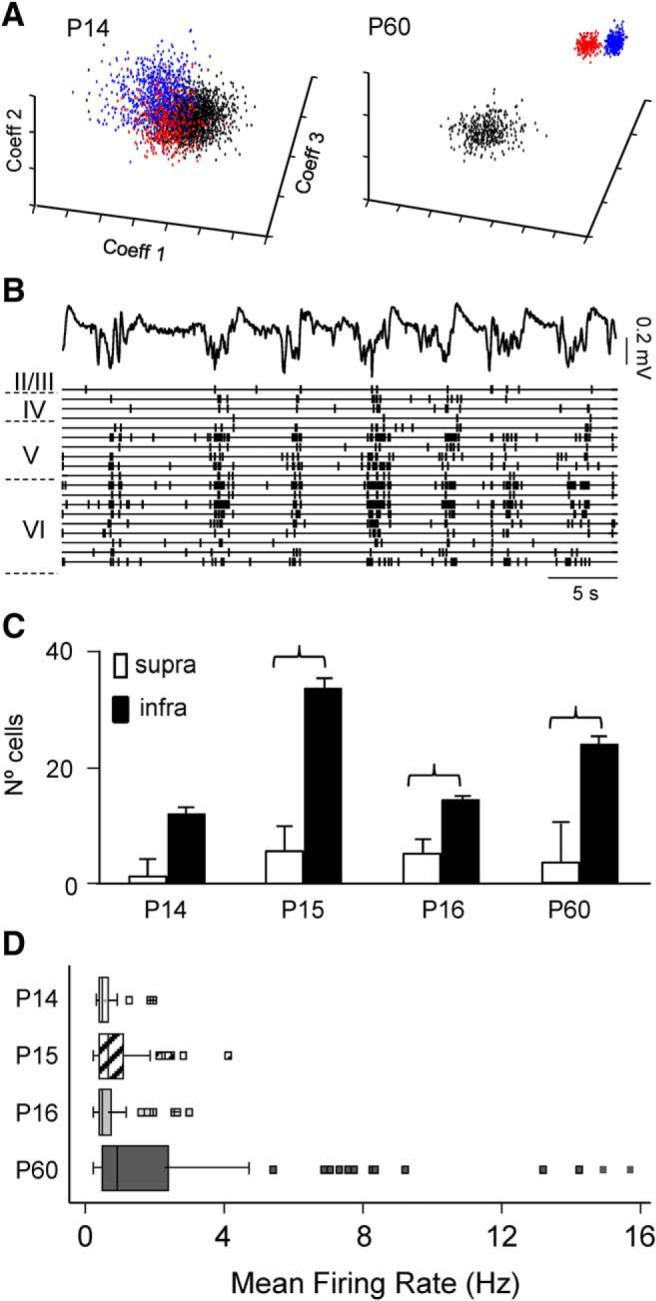
Reduced rate of spike activity in juveniles throughout all layers. ***A***, Examples of spike clouds used for the sorting of multiple units in a recording channel using the superparamagnetic method. Note the lower mean distance between three clouds of spikes obtained in one recording channel of P14 and adult representative animals. ***B***, Sample epoch showing the FP recorded in layer V and the timings of sorted spikes in different layers in a P16 specimen. Each line corresponds to a different unit. Dashed lines indicate the layer location of the units (not scaled). Note the correlation of spike firing and active periods in the FP. ***C***, Number of detected units grouped in supra and infragranular recording zones (mean and SEM, see statistics in the text, data pooled from 4 animals per age group). ***D***, Mean firing rate per age group of (data pooled across layers). Statistics as in Figure 1. Extra points are outliers.

The above data reflects postsynaptic activity, i.e., the inputs. Next, we explored the outputs (spike activity). As a general observation, the majority of unitary spikes were found in close temporal association with Up states in the FP (Fig. 8*B*). The sorting of multiple cells from a single recording channel was more challenging in juvenile animals in which spike waveform parameters displayed higher dispersion and the spike clusters found by the sorting method exhibited wider overlap (Fig. 8*A*). Given the low number of firing cells per channel we pooled data into two cortical bands of similar width and reported physiological functionality, as the infragranular and the supragranular zones. In all age groups, the infragranular zone exhibited 2–6 times more firing cells than the supragranular zone (Fig. 8*C*; *p* = 6 × 10^−7^, Mann–Whitney–Wilcoxon). In addition, P14 and P16 had a significantly smaller number of firing cells than adults (13.5 ± 3.3 and 18.4 ± 2.6 vs 27.5 ± 7.7). However, the P15 group had a high number of firing cells (39.6 ± 3.9) compared with P14 and P16 groups, and it also outnumbered the adult group.

The mean firing rate of neurons was estimated by age group (Fig. 8*D*, data pooled over all layers). Neurons in juvenile groups fired at a rate 3–6 times lower than in the adult group (e.g., P14: 0.36 ± 0.06 vs 2.16 ± 0.33 Hz). All comparisons were significant (W > 3350 and *p* < 2 × 10^−4^, *n* = 81, 198, 92, and 120 in P14, P15, P16, and adult groups, respectively, Mann–Whitney–Wilcoxon test). The P15 group again differed from P14 and P16 and had significantly higher mean rate (*p* = 0.005 and *p* = 0.008, respectively).

We then classified neurons as PI and PC units according to standard electrophysiological criteria (see Materials and Methods). The classification was somewhat less efficient in juvenile animals (63 of 120 cells in adults and 164 of 371 cells in juvenile groups, i.e., 52.5 ± 0.05% and 44.2 ± 0.1%, *n* = 5 and *n* = 15 animals, respectively). We found several notable differences among age groups and type of neuron. The half-width of extracellular spikes of putative interneurons increased moderately over the P14–P16 period and up to adults [(in ms) P14: 0.24 ± 0.02 (*n* = 5); P15: 0.35 ± 0.01 (*n* = 5); P16: 0.3 ± 0.02 (*n* = 11); Adult: 0.38 ± 0.03 (*n* = 11)], whereas the mean rate of spontaneous firing underwent a drastic 5 to sevenfold increase [(in Hz) P14: 1.53 ± 0.14; P15: 1.91 ± 0.53; P16: 1.64 ± 0.18; Adult: 10.40 ± 1.76]. By contrast, the half-width of PC spikes did not change in either group [(in ms) P14: 0.34 ± 0.02 (*n* = 27); P15: 0.34 ± 0.01 (*n* = 87); P16: 0.34 ± 0.01 (*n* = 29); Adult: 0.29 ± 0.03 (*n* = 52)], whereas the firing rate did increase, albeit to a smaller degree [∼3-fold; (in Hz) P14: 0.30 ± 0.06; P15: 0.52 ± 0.04; P16: 0.32 ± 0.06; Adult: 1.32 ± 0.59]. Overall, these data indicate that the individual neurons in juvenile individuals have not reached the mean firing characteristics of adult animals. The different ratio of excitatory to inhibitory firing neurons is compatible with found differences in the respective sets of FP generators in the two age groups.

## Discussion

We report that laminar segregation of functional inputs is far from adult-like by the time the gross anatomical lamination of the S1 rat cortex is complete. Although both juveniles and adults present similar intermittent patterns of spontaneous FP activity dominated by delta waves, these waves exhibit remarkable age-dependent laminar differences. They are raised by currents in middle cortical layers in adults, whereas in juveniles these currents are missing and it is a small generator in deep layers that generates them. The laminar distribution of evoked potentials upon peripheral stimuli also differs greatly. We propose that the synaptic pathways that become active in middle cortical layers after P16 reflect the final step of cortical circuit maturation when a number of intracortical connections start functioning and readjust their dynamic relations.

The strong overlap of neuronal elements in the cortical column promotes a severe and spatially heterogeneous cancellation of synaptic currents in the extracellular space that hinders identification of the pathways contributing to FPs. Layer-specific modulations of cortical FPs and spikes have been reported that reflect the activity of different pathways with laminar segregation (Xing et al., 2009; País-Vieira et al., 2015). To date, the most effective approach for disentangling independent sources from spontaneous FPs is the ICA, which has been validated in different brain structures and through detailed feedforward models of FPs (Makarova et al., 2011; Martín-Vázquez et al., 2016). In former studies in adult rats, we and others found FP generators with the same laminar distribution in the M1, S1FL, V2, and auditory cortices (Martín-Vázquez et al., 2018; Munro Krull et al., 2019; Torres et al., 2019). Similar results are found here for the S1HL cortex of adult animals under the same electrographic state. Such inter-area commonalities reflect the spatial organization of canonical pathways and indicate that a common subset of pathways contributes most of the FP variance across different cortices in adults.

The FP generators found show remarkably stable profiles across different days in all juvenile animals. These included the dominant one that peaked in layer VI and one gamma generator that peaked in layers II/III. The latter was among the weakest generators and yet it has spatial landmarks that closely coincide with a gamma generator in adults, thus serving as a reliable spatial reference by which the spatial jitter shown by other generators in juveniles may be interpreted as variations in the axonal coverage of inputs (Ruthazer et al., 2003) or in postsynaptic cells. In the mice barrel cortex, the postnatal growth of intrinsic connections continues up to the third week (Miller et al., 2001), although there is little information on when each becomes active. In the S1HL area of the rat, we observed (Muñoz et al., 2010) that all main circuits are already established by P14. We thus infer that few of these projections in middle layers are already functional by P14–P16.

The simple dipole of current for the layer VI generator in juveniles suggests it is generated by a single pathway. The blockade of excitatory transmission in upper/middle layers in adults blocks the broad dominant generator for delta waves and confirmed the presence of an independent smaller generator in deep layers that corresponds to the dominant generator in juveniles. We thus infer that the explosive growth of activity in middle layers after P16 is due to maturation of additional pathways. In support of this view, the broad generator is not present during cortical activated states in adults, and instead several smaller generators appear that peak close to each other throughout layers IV–VI (Torres et al., 2019). Indeed, some reports indicate that different PCs in sublayers of layer V constitute separate networks with distinct afferent and recurrent connectivity and extracortical targets (Morishima et al., 2011). In addition, thalamocortical cells produce combined or separate excitation in layers IV and VI, which indicates different types of afferent cells projecting to a common target area (Swadlow et al., 2002). It is thus possible that inputs to the cortex from a common thalamic origin have different developmental schedules, while only when they gain sufficient coherence during Up states in adulthood the ICA fails to separate them.

Regarding the small generators in juveniles that display spatial jitter and were not present in adults, we cannot resolve if they reflect activity in the same pathways that undergo spatial readjustment after P16 or alternatively, whether there is a different set of pathways contributing to FPs in each age group. One possible explanation is that the axonal terminal field of some pathways in PCs modifies after P16, either by synapse pruning, synaptogenesis, or activation of formerly silent synapses (Larsen and Callaway, 2006; Romand et al., 2011; Huang et al., 2015; Blanquie et al., 2017). Alternatively, some pathways may form their initial contact on S1HL target neurons by P14–P16 and later they colonize dendritic portions and laminae not yet used (Ruthazer et al., 2003). For instance, it is plausible that associational connections between different cortices become functional only after the specific cortices have mature intra-columnar networks and once the somatotopic maps of thalamocortical inputs have completed, in line with a developmental schedule for the stabilization of feedforward and associative cortical circuits proposed by Singer (1995).

Another possibility is that FP generators in juveniles reflect the spiking activity of pathways that will become less active in adults as others take over. It has previously been indicated that the firing properties of main cortical cell types stabilize after P10 in cortex *in vitro* (Picken Bahrey and Moody, 2003), but these can hardly be extrapolated to *in vivo* spontaneous firing. Indeed, we find that the putative interneuron population drastically increases the firing rate from P16 to adulthood, which also suggests a notable impact on the overall balance of excitation and inhibition. This is in line with the anatomical and neurochemical studies indicating a progressive and prominent increase in the excitatory to inhibitory synapse ratio that occurs in a layer-specific manner (DeFelipe et al., 1997). Additional experiments are required to increase our knowledge regarding developmental firing patterns of specific interneuron subtypes.

The relation between intra-columnar segments is highly dynamic in adults due to intracortical loops and modulatory pathways (País-Vieira et al., 2015; Reyes-Puerta et al., 2015). Before the peripheral input begins, there are fewer firing neurons. Layer-specific refinement of spike firing has been reported in other cortices up to P28 in mice (van der Bourg et al., 2017) and we show here that it continues until adulthood in the rat as well. The different ranges of spike activity transferred among specific cell subtypes in the cortical column (Ushimaru and Kawaguchi, 2015) thus appear to vary from juvenile until adulthood. In relation to this, we find significant cross-correlations in ∼50% pairs of generators, which indicate a significant amount of correlated firing of the two afferent neuron populations that originate them. Eventually, the identification of the pathways giving raise to each cortical FP generator will permit this promising observation to be extended revealing how specific pathways gradually change their activity and computational function until adulthood, which is unlikely to be achieved solely through unitary activity as the spikes lack specification of the synaptic inputs that originate them.

The age-dependent spatial differences found for spontaneous FP activity are consistent with the different distribution of peripherally-evoked FPs and CSDs. Analogous differences between spontaneous and evoked activities have been reported in laminar analysis of spike firing in other cortices (Reyes-Puerta et al., 2015, 2016; Ushimaru and Kawaguchi, 2015). We also found different profiles for tactile or low-intensity electrical peripheral stimuli in both groups of age, possibly because of the dissimilar activation of the lemniscal and paralemniscal ascending pathways that reach the cortex in different layers (Bureau et al., 2006; Moxon et al., 2008; Wimmer et al., 2010; País-Vieira et al., 2015). However, other remarkable differences are difficult to explain, such as the inverted dipole and the different locations of the earliest response in evoked potentials, which strongly indicates different inputs, possibly targeting different PCs. Some of the present data reflect the poor myelination in P14–P16 animals, such as the much longer latency of evoked responses. Part of this delay appears to be due to myelination of thalamocortical fibers (Salami et al., 2003).

### Concluding remarks

Most developmental oscillatory activity is driven from the periphery (e.g., retinal waves or whisker twitches) and it is known to have a trophic action leading to circuit maturation in which the intrinsic properties and connectivity of neonatal cortical cells appear to be specifically optimized for developmental functions (Moody and Bosma, 2005). In this regard, we envisage the differences found in P14–P16 juveniles and adults as a yet unfinished global process of maturation that shows up as a different laminar organization of active synaptic inputs. It follows that a precise intra-columnar organization of functional connections between neuron subtypes is not yet complete, and therefore cortical processing of peripheral input would not be ready for cognitive and behavioral purposes. We propose that the correct spatial organization of FP activity can be used as a developmental marker for functional maturity of specific cortical areas.
